# Diversity of *Mycobacterium tuberculosis* in the Middle Fly District of Western Province, Papua New Guinea: microbead-based spoligotyping using DNA from Ziehl-Neelsen-stained microscopy preparations

**DOI:** 10.1038/s41598-019-51892-5

**Published:** 2019-10-29

**Authors:** Vanina Guernier-Cambert, Tanya Diefenbach-Elstob, Bernice J. Klotoe, Graham Burgess, Daniel Pelowa, Robert Dowi, Bisato Gula, Emma S. McBryde, Guislaine Refrégier, Catherine Rush, Christophe Sola, Jeffrey Warner

**Affiliations:** 1 Australian Institute of Tropical Health and Medicine, James Cook University, Townsville, Queensland Australia; 2 College of Public Health, Medical and Veterinary Sciences, James Cook University, Townsville, Queensland Australia; 3 Institut de Biologie Intégrative de la Cellule (I2BC), CEA, CNRS, Université Paris-Sud, Université Paris-Saclay, Gif-sur-Yvette, Orsay, France; 4 Balimo District Hospital, Balimo, Western Province Papua New Guinea; 5 Department of Medicine, Royal Melbourne Hospital, University of Melbourne, Melbourne, Victoria Australia; 6 Present Address: National Animal Disease Center, Agricultural Research Service, United States Department of Agriculture, Ames, 50010 IA USA

**Keywords:** Molecular biology, Tuberculosis, Epidemiology, Phylogenetics

## Abstract

Tuberculosis remains the world’s leading cause of death from an infectious agent, and is a serious health problem in Papua New Guinea (PNG) with an estimated 36,000 new cases each year. This study describes the genetic diversity of *Mycobacterium tuberculosis* among tuberculosis patients in the Balimo/Bamu region in the Middle Fly District of Western Province in PNG, and investigates rifampicin resistance-associated mutations. Archived Ziehl-Neelsen-stained sputum smears were used to conduct microbead-based spoligotyping and assess genotypic resistance. Among the 162 samples included, 80 (49.4%) generated spoligotyping patterns (n = 23), belonging predominantly to the L2 Lineage (44%) and the L4 Lineage (30%). This is consistent with what has been found in other PNG regions geographically distant from Middle Fly District of Western Province, but is different from neighbouring South-East Asian countries. Rifampicin resistance was identified in 7.8% of the successfully sequenced samples, with all resistant samples belonging to the L2/Beijing Lineage. A high prevalence of mixed L2/L4 profiles was suggestive of polyclonal infection in the region, although this would need to be confirmed. The method described here could be a game-changer in resource-limited countries where large numbers of archived smear slides could be used for retrospective (and prospective) studies of *M. tuberculosis* genetic epidemiology.

## Introduction

Papua New Guinea (PNG) is one of the ten high tuberculosis (TB) burden countries worldwide with a TB notification rate of 333 per 100,000 people (n = 28,244) in 2016^1^. The World Health Organization (WHO)-estimated incidence rate was 432 cases per 100,000 population (95% CI 352 to 521) in 2017, accounting for 36,000 new cases and 4,300 deaths^2^. The TB burden can be even higher in rural areas of PNG, with estimated incidence rates of 1,290 cases per 100,000 population in Gulf Province and 550 cases per 100,000 in Western Province (WP)^3,4^. Considering that HIV (a major driver of TB in sub-Saharan Africa) accounts for only about 5–10% of TB cases in PNG, these estimated incidence rates are remarkably high^4^. PNG is also considered to have a high burden of rifampicin-resistant TB (RR-TB) and multidrug-resistant TB (MDR-TB), defined as resistance to at least rifampicin (RIF) and isoniazid (INH)^2^. In 2017, the proportion of RR-TB and MDR-TB (referred to as MDR/RR-TB) was estimated to be 3.4% of new TB cases and 26% of retreatment cases^2^.

Comparing the genetic diversity of *Mycobacterium tuberculosis* (*Mtb*) within and between different populations provides insights into TB dynamics at different levels, and may help improve control strategies developed by national TB control programs. Seven lineages of human adapted *M. tuberculosis* complex (MTBC) have been identified^5^. The so-called “modern lineages” (Lineages 2, 3 and 4) are widespread, while the “ancient” (Lineages 1, 5 and 6) show spatial heterogeneity^6^. Modern lineages have been suggested to have increased virulence or transmissibility, which may have evolved as a response to co-evolutionary interactions with particular human populations, or to mass BCG vaccination and use of antituberculosis drugs^7^. In PNG, molecular epidemiology studies based on MTBC clinical isolates are scarce. Two studies, published in 2012^8^ and in 2014^9^ investigated the diversity of *Mtb* in different PNG provinces with cultured samples using spoligotyping, a method using the CRISPR locus (clustered regularly interspaced short palindromic repeats) as a target for *Mtb* subtyping^10^*. Mtb* isolates originating from adult TB patients from Madang and surrounds belonged predominantly to Lineage 4 and Lineage 2, and 44% of isolates were molecularly clustered, suggesting active *Mtb* transmission in the community^8^. Spoligotyping of *Mtb* isolates from Goroka (Eastern Highlands), Madang (Madang Province) and Alotau (Milne Bay) revealed L4, L2, and L1 Lineages as the main *Mtb* lineages at all three sites^9^. More recent studies also used variable number of tandem repeats (VNTR) and whole genome sequencing (WGS) to understand the genetic diversity of circulating strains, and revealed drug resistance-associated mutations on Daru Island, in the South Fly District WP, PNG^11,12^. In Daru, a cluster of MDR/RR-TB (n = 95) was identified as belonging to a single L2/Beijing sub-lineage 2.2.1.1^12^. Nevertheless, the diversity of *Mtb* in PNG remains largely unexplored, and no information is available from the Middle Fly District WP.

Overall, facilities for culturing clinical isolates and testing them for drug susceptibility are lacking in PNG. In the Middle Fly District WP, the catchment area for Balimo District Hospital (BDH) primarily includes the population of the ‘Balimo Urban’ and ‘Gogodala Rural’ local level government (LLG) areas, and to a lesser degree the ‘Bamu Rural’ LLG. This area is hereafter referred to as the “Balimo/Bamu region”. BDH does not have facilities for performing *Mtb* culture and is not equipped with a GeneXpert (Cepheid) instrument, so TB diagnosis relies primarily on clinical presentation and direct sputum smear microscopy, based on the PNG National Tuberculosis Management Protocol^13–15^. DNA extracted from Ziehl-Neelsen (ZN) stained sputum smears has already proven useful in molecularly confirming the microscopy diagnosis of TB in this region of PNG^14^, and spoligotyping has been undertaken successfully on DNA extracted from cultures^10,16^, from decontaminated sputa^17^, as well as from ZN-stained sputum smears^18–21^. Direct sputum smear microscopy is routinely performed in diagnostic laboratories worldwide, including in developing countries, and stained slides can be stored and transported at room temperature, which makes them a prime target for spoligotyping of *Mtb* samples collected in remote settings^21^. Spoligotyping has been historically performed by reverse hybridization on a nylon membrane^22^ but microbead-based systems improve throughput and sensitivity^10,23^. We performed microbead-based spoligotyping and molecular analysis of ZN-stained sputum smears collected in the Balimo/Bamu region in the Middle Fly District WP, PNG, in order to (i) assess the efficacy of the spoligotyping method when using archived samples collected under routine programmatic conditions in a remote location, (ii) analyse the genetic diversity and distribution of *Mtb*, and (iii) identify RIF-resistant TB among newly diagnosed TB patients.

## Results

### Clinical sample set

A total of 345 smeared sputa were collected from June 2012 to March 2014, of which 206 showed evidence of mycobacterial DNA based on combined TaqMan qPCR assays (see^14^ for details). Out of those 206 qPCR-positive samples, the spoligotyping and resistance analyses focused on the ones with the lowest *Cq* values after qPCR, with a cutoff of 36 chosen from preliminary spoligotyping results. Samples included in the analyses (n = 162) are summarised in Table 1, with their detailed microscopy results. The excluded samples (n = 44; *Cq* > 36) were mostly negative in microscopy (36 negative, 3 scanty, 5 unknown).

**Table 1:** Detailed microscopy results of the included qPCR-positive sputum smears (n = 162), and of the successfully spoligotyped samples. ‘Mixed’: spoligotype pattern interpreted as an intermediate L2/L4 lineage.

Microscopy results	qPCR-positive	Spoligotyping	Spoligotyping-based lineage assignations
3+	47	42	16 L4; 23 L2; 3 mixed
2+	15	15	2 L4; 6 L2; 6 mixed; 1 L1
1+	22	9	3 L4; 1 L2; 5 mixed
scanty	7	3	1 L4; 2 mixed
negative	54	6	1 L4; 1 L2; 4 mixed
unkown	17	5	1 L4; 4 L2
Total	162	80	24 L4; 35 L2; 20 mixed; 1 L1

### Genotypic diversity of the clinical isolates

Spoligotyping of *Mtb* was successfully conducted in 49.4% (80/162) samples (Table 1). Interpretable spoligotyping results were obtained for 92% (57/62) of the sputum smears showing ‘3+’ and ‘2+’ microscopy results; the spoligotyping success dropped to 41% (12/29) for the sputum smears with ‘1+’ and ‘scanty’ microscopy results. Three of seven worldwide reported *Mtb* lineages were detected in the Balimo/Bamu region (Table 1). Among the 80 independent samples with successful genotyping, modern Lineages L2 and L4 were most prevalent, representing 44% (35/80) and 30% (24/80) of the samples respectively. In contrast, ancient L1 Lineage was very rare (1%; 1/80). Nine spoligotype profiles were atypical (only one reported in SITVIT2, one reported in Madang, PNG, and all giving contradictory labellings with TBminer), and careful exploration of raw signals showed intermediate values for several spacers, especially sp35 and sp36 that are absent in L4 Lineage and present in L2 Lineage. These mixed patterns likely corresponded to mixed infections of L2 and L4 strains as reported in other studies (see Discussion) and represented 25% (20/80) of the samples (Table 1). When excluding ‘mixed’ samples, the prevalences per lineage were 2% (1/60) for L1, 58% (35/60) for L2, and 40% (24/60) for L4.

**Figure 1:**
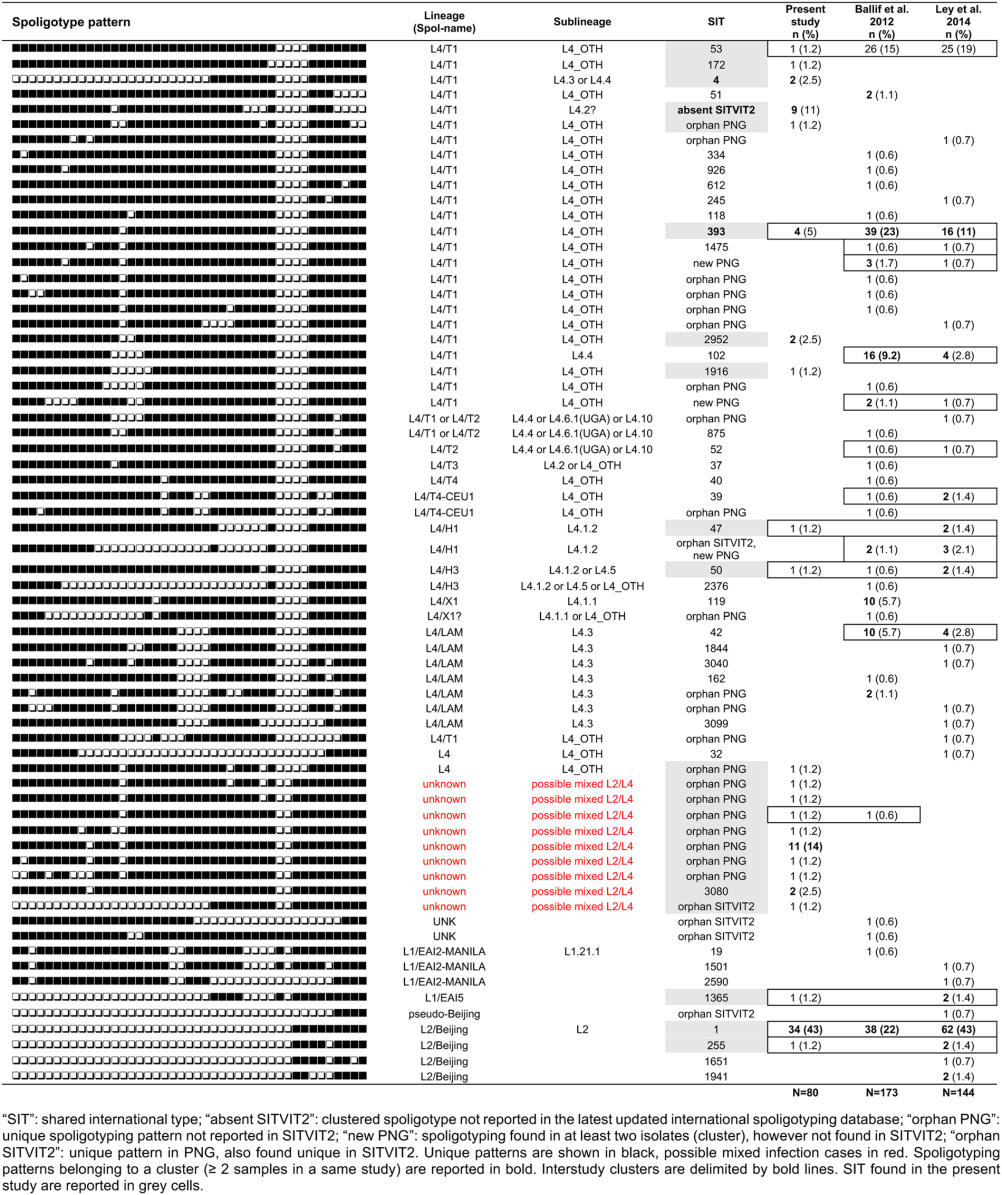
Spoligotype patterns and lineages detected in our study and in previous studies from different provinces in PNG. The present study focuses on patients from the Balimo/Bamu region (Middle Fly District of Western Province), all diagnosed at the Balimo District hospital. The Baillif et *al*. 2012 study^8^ focuses on patients from Madang Province, East Sepik Province and “other”, all diagnosed at the main hospital of Madang Province (Modilon General Hospital). The Ley et *al*. 2014 study^9^ focuses on patients from Madang (Madang Province), Goroka (Eastern Highlands Province) and Alotau (Milne Bay Province).

Twenty-three different spoligotyping patterns belonged to 13 different families and five clusters with two or more samples (Fig. 1). The largest clusters were L2/Beijing SIT1 (n = 34), one new L4 pattern (likely L4.2/Ural1 according to TBminer) (n = 9), and one pattern labelled as ‘mixed’ (n = 11). Of the 23 patterns identified, eight patterns (n = 44 samples) have already been described in PNG, including four patterns described in the two previous PNG studies^8,9^ as well as ours: L2/Beijing SIT1, L4/H3 SIT50, L4/T1 SIT53 and L4/T1 SIT393 (Fig. 1). The other 15 patterns (n = 36 samples) were “new” in PNG. Of note, eight patterns were detected in the two previous PNG studies^8,9^ but were not identified in the Balimo/Bamu region, including L4/X1 SIT119 and L4/LAM9 SIT42 that each accounted for 6% of spoligotypes in Madang^8^.

### Spatial analysis of *Mtb* lineages in PNG

**Figure 2:**
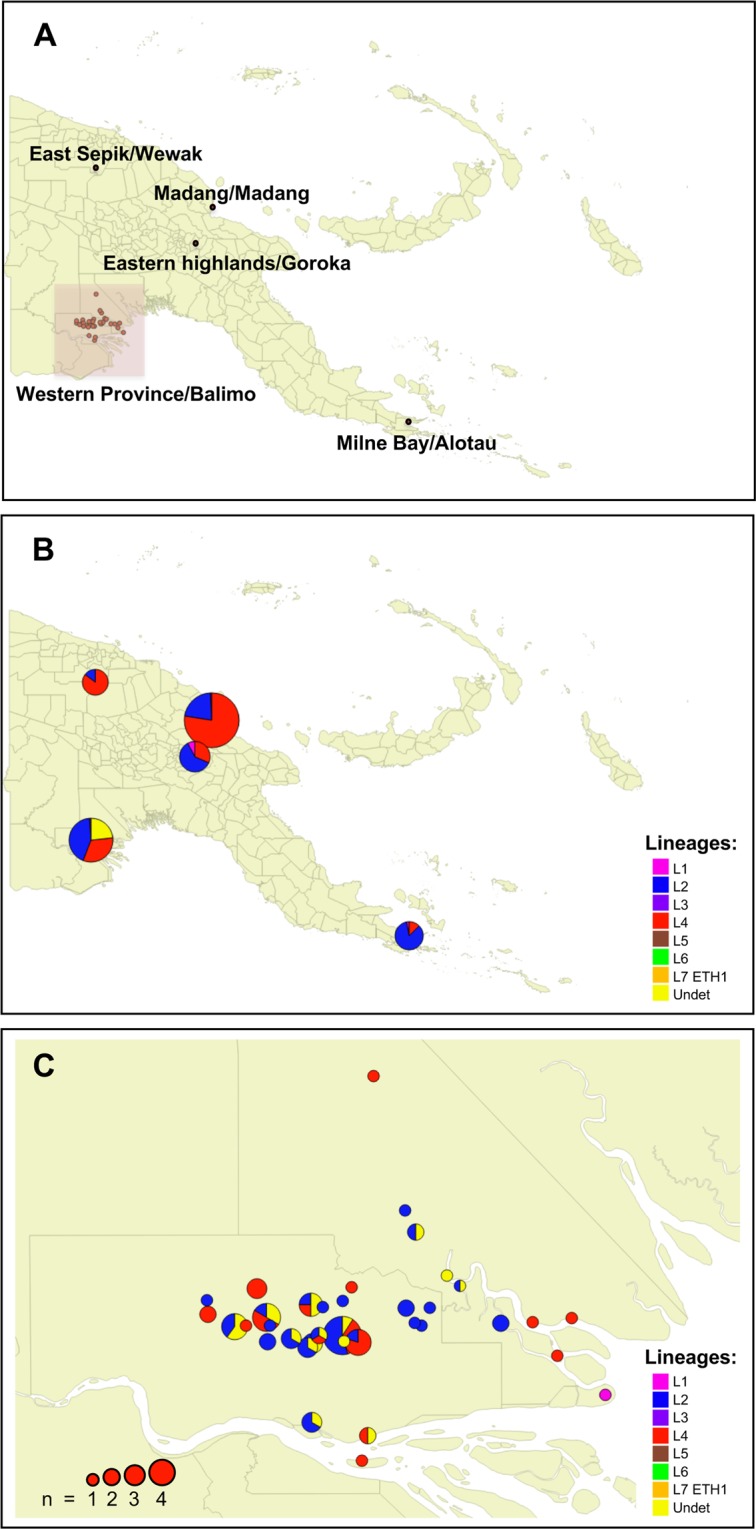
Spatial distribution of *Mtb* spoligotyping-defined lineages in Papua New Guinea. (**A**) Localization of previous study settings, as well as the present study in the Balimo/Bamu region, Middle Fly District of Western Province (red square). (**B**) Distribution of *Mtb* lineages at the regional level in Goroka, Alotau, Madang^9^, Madang Province and East Sepik Province^8^ and the Balimo/Bamu region (this study). (**C**) Distribution of *Mtb* lineages at the subregional level in the Balimo/Bamu region. Size of circle is proportional to the number of TB cases (n). Local level government (LLG) areas are delimited in grey lines, with TB cases in (**C**) located in the Gogodala Rural (southwest, including the Balimo Urban LLG) and Bamu Rural (northeast) LLGs. Undet: undetermined (including intermediate L2/L4 patterns; see main text for further explanation).

Figure 2 shows the distribution of *Mtb* diversity according to previous investigations in PNG (Fig. 2B) and the present study (Fig. 2B,C). L2 and L4 Lineages were present in all PNG settings, while L1 Lineage was detected in samples from three locations out of the five regions investigated, including the Balimo/Bamu region where it was detected in one sample (Fig. 2C). However, the relative percentage of each lineage varied between locations at the national level (Fig. 2B) as well as at the regional level (Fig. 2C). In the present study, the biggest spatial cluster (n = 8) corresponds to the town of Balimo (Fig. 2C).

### RIF-resistance associated mutations

RIF-resistance was assessed by sequencing a 147-bp region of the *rpoB* gene (including RRDR). Out of the 162 qPCR-positive sputum smears included in the analyses, 116 (71.6%) were successfully sequenced (Table 2). Mutations were identified in 7.8% (95% CI 2.9–12.6%) of the successfully sequenced samples, and all corresponded to the *rpoB* S450L codon mutation (Table 2). Of note, one sample that tested positive for mycobacterial DNA based on qPCR resulted in a *rpoB* sequence originating from *Pseudomonas aeruginosa*. Considering that this sample showed a late positive qPCR (*Cq* 33.3 and 35.5), a negative microscopy result, but a ‘1 +’ microscopy result on another slide from the same patient sampled the previous month, we believe this patient to be a true but low MTBC positive and a mixed sample containing MTBC and *Pseudomonas*.

**Table 2:** Summary of sequencing results for the *rpoB* amplicons, including nucleotide and codon mutations.

*rpoB* result	n	% of 162	% of 115
WT	100	61.7%	87.0%
Probable WT^a^	6	3.7%	5.2%
S450L^b^ (C1349T)	9	5.6%	7.8%
N/A	46	28.4%	—
Not matched to ref	1	0.6%	—
Total	162	100%	100%

n: number of samples; WT: wild-type (no mutations identified); N/A: not available (PCR amplification or sequencing failed); Not matched to ref: obtained sequence not matching the MTB H37Rv reference genome; ^a^Few nucleotides missing at positions other than those associated with resistance, otherwise matching wild type; ^b^Codon mutation located within the RRDR of the *rpoB* gene, numbered according to the system based on MTB H37Rv.

Among the 79 sputum smears with both *rpoB* and spoligotyping results, 72 were *rpoB* wild type and seven were S450L mutants, with all the RIF-resistant samples belonging to the L2/Beijing Lineage (Fig. S1). Drug resistance was thus linked with Lineage 2 (Fisher exact test p = 0.003), as already observed in a study conducted at Modilon General Hospital, Madang Province^8^.

## Discussion

The spoligotyping method is known to have limited discriminatory capacity due to the fact that it targets only a single genetic locus, covering less than 0.1% of the *Mtb* genome, and when used alone, is not sufficient for epidemiological linking studies^24^. Nevertheless, spoligotyping allows identification of most if not all genotypes of significant, clinical, and epidemiological relevance, such as the “Beijing” genotype^24^. In this study, we used spoligotyping to describe the genetic diversity of *Mtb* from patients presenting at BDH, originating from the Middle Fly District WP, PNG. We show a wide diversity of spoligotype patterns in the Balimo/Bamu region, and confirm the presence of the modern L2 and L4 Lineages, with a single ancient L1 Lineage case, originating from the Bamu LLG. We also analysed drug-resistance associated mutations in the *rpoB* gene, and identified 7.8% of the successfully sequenced samples showing RIF resistance, all being L2/Beijing Lineage isolates.

Populations in the Highlands are the oldest settlers of PNG, as shown by human genetic studies^25^, and have been isolated from the outside world for much longer than the populations in coastal sites. For this reason it was expected that evolutionary ancient lineages of *Mtb* (e.g. L1 Lineage) would be found in the low density populations of the Highlands, as opposed to modern lineages of *Mtb* (L2 and L4 Lineages) that would be found in the high density populations of the coastal regions^9^. However, a recent human genetic study determined that the genetic differentiation between populations from highland and lowland PNG does not seem to be as significant as previously thought^26^, and no statistically significant difference in the prevalence of L1 Lineage could be found in Goroka, Eastern Highlands, compared to coastal regions^9^. In the end, the pattern of dominance of modern lineages over ancient L1 Lineage seems consistent across PNG, with the 2% of L1 Lineage found in the Balimo/Bamu region not dissimilar to the 0.6% (1/173) to 8% (3/38) observed in the North Coast (East Sepik, Madang), Goroka or Alotau^8,9^. This pattern is strikingly different from what has been described in neighbouring South-East Asian countries. In Western New Guinea (WNG), i.e. the Indonesian part of the island, the same three lineages have been reported, but with a higher proportion of L1 Lineage (33.7%)^27^. This is also true in other countries of the region, with L1 Lineage representing 30.2% of samples in Makassar, Indonesia^28^, 56.4% in Malaysia^29^, and 36.3% in southern Vietnam^30^. This might be due to different importation histories of TB strains in PNG compared to the rest of South-East Asia. For example, it has been suggested that ancient TB lineages might have been replaced early on by modern and more successful lineages with the arrival of Europeans in PNG in the late 19^th^ century^31^.

In L4 Lineage, Latin-American and Mediterreanean (LAM) families (Lineage 4.3) were the most prevalent in WNG, as compared to PNG where other L4 families were found in high proportion in most of the locations. Interestingly, L4/SIT393 is reported in the three independent studies performed so far in PNG. L4/SIT393 is characterized by the absence of spacer sp14, a character that has not yet been shown to be monophyletic. A total of 60 cases have now been reported in PNG (Fig. 1), whereas until now, only 36 cases were described worldwide in SITVIT2^32^. The largest and best collection of L4 Lineage characterized so far in PNG has been described by Stucki and colleagues^33^ that report, within the L4 Lineage, up to 40% of L4.10/PGG3 (Principal Genetic Group 3) strains, characterized by the katG463-CGG (Arg) plus gyrA95-AGC (Ser) combination and the Rv1501 C- > A allelic change^34,35^. We suggest that L4/SIT393 isolated in PNG could belong to the L4.10/PGG3 lineage. Future work should focus on SNP characterization of these isolates in PNG, and related analyses should shed interesting light into diversity at the sublineage level.

An interesting result from our analysis was the likely identification of many mixed (or polyclonal) infections, which are infections with several different genotypes/strains of *Mtb* in a single patient^36^. In our study, many spoligotype patterns could be assigned as MANU2 in SITVIT2, but have been assigned as “mixed” patterns in our analysis, with strains harboring superimpositions of L2 and L4 patterns (Table 1), and seven orphans showing similar patterns. It has been suggested that mixed infections in clinical specimens can challenge TB spoligotyping and sequencing techniques, and might have been the cause of false spoligotypes previously reported^37,38^. MANU2 spoligotype has been suggested to be one of the five genotypes derived from mixed infections in clinical specimens^39^. Studies showing the prevalence of MANU2 and discussing a link between MANU2 and mixed infection have been conducted in Iran^37^, Mozambique^40^, and Sudan^41^. The study in Mozambique concluded that some of the MANU2 genotypes could be derived from mixed infections of L2/Beijing and L4/T1, or L2/Beijing and L4/T2. Further studies on samples showing “mixed” patterns are required to more fully establish whether polyclonal infections (from either coinfection or superinfection) are present in the Balimo/Bamu region of PNG. This information may be epidemiologically relevant as it may indicate high contact rates of individuals with diverse *Mtb* strains.

Typical L2/Beijing strains are reported at high frequency worldwide and have been associated with drug resistance and increased transmissibility^42,43^. Sequencing of a 147-bp *rpoB* region identified seven samples as RIF-resistant, all presenting the *rpoB* S450L codon mutation. This is in agreement with previous findings in the Balimo/Bamu region WP^44^ and in South Fly District WP^12^ and other provinces in PNG^8,9^. All RIF-resistant samples belonged to the L2/Beijing Lineage, as found in Vietnam^30,45^. Molecular epidemiological studies have suggested that certain *Mtb* genotypes are more successful than others, including some L2/Beijing genotypes that have a higher fitness and have evolved unique pathogenic characteristics^42,46,47^. Phylogenetic analysis has provided evidence that the more recently diversified strains (so-called modern/typical Beijing L2.3) are adapted to spread and cause disease, compared to other sublineages (so-called ancient/atypical Beijing L2.2 or proto-Beijing, L2.1)^48^. On Daru Island in the South Fly District WP, a drug-resistant TB outbreak was recently characterized using WGS and was found to be driven by a Beijing L2.2.1.1 strain^12^, corresponding to a modern Beijing L2.3 in the latest classification from Liu and colleagues^48^. Unfortunately, our study did not provide any information at the sublineage level regarding the L2/Beijing strains. To complement our spoligotyping, further genomic studies of this sublineage in the Balimo/Bamu region of PNG will be needed to assess the presence of super-spreaders, association with drug resistance, or any other genetic signature of epidemiological interest.

Our study confirms the limitation that clean spoligotyping results can be obtained mostly from AFB 3+ and 2+ slides. Nevertheless, the collected information about genetic diversity is invaluable for better understanding the epidemiology of TB, and archived smear slides are of sufficient quality to collect such data, as seen in this study. It is likely that prior targeted enrichment techniques may have improved our direct genotyping results from sputum smears^49,50^. However, such methods are not yet appropriate to local processing of samples. Before more sensitive methods can be introduced in countries such as PNG, huge numbers of archived smear slides could be used for retrospective and prospective studies of *Mtb* diversity.

In summary we demonstrate that, in countries where conservation of samples might be an issue, and/or hospital laboratories do not have the capacity for culture or Xpert MTB/RIF-based diagnosis of TB, uncultured biological material could be used to obtain rich genomic information using archived sputum smears. Increasing local *in vitro* diagnostic capacities using devices such as the GeneXpert (Cepheid, USA) is important to increase awareness and improve public health. Alternatively, research on efficiency of new lab-on-chip devices, that should be as simple and inexpensive as possible to be used in level 1 laboratories and run as close as possible to the bedside of the patient, could be another way to improve TB control in rural regions of PNG.

## Methods

### Study setting and sample collection

BDH is located in the town of Balimo in the Middle Fly District WP, PNG. Passive case detection for TB is conducted at BDH among individuals self-presenting for health care. Presumptive TB cases presenting at BDH were from the Balimo Urban, Gogodala Rural, and Bamu Rural LLG areas, i.e. three of the five LLGs belonging to the Middle Fly District WP. Sputum samples are collected routinely for diagnostic purposes from patients with clinical signs of TB, and are ZN-stained and examined for AFB (acid-fast bacilli) by light microscopy, with slides then archived and stored. Each sputum sample was smeared on several slides, with up to five slides per sputum sample (see^14^ for details). Archived slides were transferred to James Cook University, in Townsville, Australia, for DNA extraction from the sputum smears.

### DNA extraction and detection of *Mycobacterium* species

DNA extracts were obtained using a modified version of a previously described Chelex DNA extraction method in biosafety level 2 laboratories^21^; the modified protocol has been published elswhere^14^. The presence of MTBC in the extracted DNA was analyzed using a TaqMan real-time PCR^51^ and the molecular diagnosis results have been published elsewhere^14^. Regarding duplicate sputum smears (i.e. originating from a single patient), only the smear showing the lowest *Cq* value was selected for further analysis. DNA aliquots were sent to the Institute for Integrative Cell Biology (Gif-sur-Yvette, France) for high-throughput spoligotyping.

### Microbead-based spoligotyping

Microbead-based hybridization was performed as previously described^10,23^ using the ‘TB-SPOL’ kits purchased from Beamedex (Beamedex, Orsay, France; www.beamedex.com). Detection was performed using a Luminex® 200 platform (Luminex Corp, Austin, TX, USA) and XPONENT software for LX100/LX200 (version 3.1.871.0). Interpretation of raw data was undertaken jointly by two experts (CS, GR) who idependently assessed spoligotyping patterns as previously described^52^. When no clear-cut interpretation was possible, typing was considered as failed; in other cases, intermediate quantitative signals for some spacers only allowed categorization of the samples as “mixed patterns” (see Discussion). Individual spoligotyping patterns were further compared with the International Spoligotyping Database (SITVIT2) of Pasteur Institute of Guadeloupe (http://www.pasteur-guadeloupe.fr:8081/SITVIT2/indexfr.jsp), the latest updated release of SITVITWEB database^32^. Shared International Types (SIT) and spoligotype lineages and families were assigned according to signatures provided in SITVIT2 and to the latest genome-based lineage designations, i.e. L1-L7 Lineage labels and sublineage labels when possible, according to^53^ and^34^. Spoligotyping patterns absent in SITVIT2 were reported as “orphans”, as well as the patterns already described as “orphans” in the SITVIT2 database with no assigned SIT. Additional exploration of the family to which strains belonged was performed using TBminer (https://info-demo.lirmm.fr/tbminer/)^54^. This tool provides assignation according to different classifications that largely overlap. Contradictory assignations between the different classifications are indicative of either newly described subfamilies or of artefacts in the genotyping data (unpublished results). Spoligotyping from smear slides has previously shown better results with AFB-positive sputa quantified as '2+' and '3+' because of higher DNA loads^55^. However, instead of using microscopy results, we took into account *Cq* values obtained from qPCR targeting MTBC to select samples showing high DNA loads (see^14^ for detailed qPCR results). The *Cq* cutoff to be used was chosen from preliminary spoligotyping results.

### *rpoB* mutations analysis

MDR/RR-TB was inferred from drug resistance-associated gene mutations of *Mtb*, focusing on the well-described *rpoB* gene. A qPCR was used to identify mutations in the *rpoB* gene using the DNA extracted from the TB clinical samples as a template. The *rpoB* primer set generating a 147-bp product was designed in-house and synthesized by Macrogen Inc. (Republic of Korea): Mtb-rpoB-147-F (5′-ATC AAG GAG TTC TTC GGC ACC AG-3′) and Mtb-rpoB-147-R (5′-CAC GTC GCG GAC CTC CAG-3′). Each qPCR reaction (20 μL) contained 1X GoTaq qPCR Master Mix (Promega, Madison, USA), 0.8 μM each of forward and reverse primer, and 2 μL of DNA template. The positive control used MTB H37Rv (GenBank: AL123456.3). Thermal cycling was as follows: hold at 95 °C for 2 min, followed by 45 cycles of 95 °C for 5 s and 60 °C for 15 s. Melting curve analysis was performed at a linear temperature transition rate of 0.1 °C/s from 70 to 95 °C. The qPCR assays were optimized and undertaken on a Rotor-Gene qPCR machine (Rotor-Gene software version 2.0.2.4). Samples that were reactive (*Cq* ≤ 38) were sequenced in both directions (Macrogen Inc., Republic of Korea). Sequence chromatograms were analysed using Geneious R10 (Biomatters Limited, Auckland, New Zealand), with the consensus sequences of each sample aligned with and compared to the MTB H37Rv reference sequence (GenBank: AL123456.3) using the “Map to Reference” Geneious tool. The consensus numbering system used for the RIF-resistance associated *rpoB* gene mutations is according to that described previously for MTB H37Rv^56^.

### Mapping the data

Patient data was obtained from the BDH TB patient and laboratory registers, with patient locations identified as the first residential address recorded. Each patient’s location was matched to a census unit, based primarily on PNG census data obtained from the PNG National Statistical Office, or alternatively from the 2012 and 2017 PNG government election polling schedules^57,58^. Latitude and longitude coordinates were obtained from the PNG National Statistical Office and census data, based on census unit-level data for villages, and averaged coordinates of multiple census units for Balimo and larger villages where the precise census unit was unknown. Instances of alternate local names were checked and confirmed locally. Out of the 162 included samples, the residential address was not able to be determined for 14 (address absent from the register for 12 patients; unknown villages ‘Sarau’ and ‘Owa’ for two patients). Maps were created using a Mac OSX Version of QGIS v2.18 Las Palmas de Gran Canaria^59^ (available from: http://www.kyngchaos.com/software/qgis). A licence-free PNG map with administrative areas (level 1) was downloaded as a shapefile from http://www.diva-gis.org/gdata.

### Ethics statement

Ethics approval was obtained from the PNG Medical Research Advisory Council and registered under the reference MRAC No. 17.02. The information sheet and written informed consent forms were available where required. However, the need for written informed consent was waived by the Ethics Committee due to generally low levels of literacy, and verbal consent was obtained in the majority of the cases. The study was conducted with the permission and support of the Middle Fly District Health Service and the Evangelical Church of PNG Health Service, and at all times permission was obtained prior to sampling activities.

## Supplementary information

Figure S1

## Data Availability

All data generated or analysed during this study are included in the published article.
