# Supplementary material for: Diversity of Mycobacterium tuberculosis in the Middle Fly District of Western Province, Papua New Guinea: microbead-based spoligotyping using DNA from Ziehl-Neelsen-stained microscopy preparations
Source: Sci Rep. 2019 Oct 29;9:15549. doi: 10.1038/s41598-019-51892-5 (PMC6820861; doi:10.1038/s41598-019-51892-5)
Supplement: Supplementary file 1 — Figure S1 [file 41598_2019_51892_MOESM1_ESM.pdf]

Supplementary Material 1

Diversity of *Mycobacterium tuberculosis* in the Middle Fly District of Western Province, Papua New Guinea: microbead-based spoligotyping using DNA from Ziehl-Neelsen-stained microscopy preparations

Vanina Guernier-Cambert[1*¤], Tanya Diefenbach-Elstob[1,2], Bernice J. Klotoe[3], Graham Burgess[2], Daniel Pelowa[4], Robert Dowi[4], Bisato Gula[4], Emma S. McBryde[1,5], Guislaine Refrégier[3], Catherine Rush[1,2], Christophe Sola[3**], Jeffrey Warner[1,2**]

[1] Australian Institute of Tropical Health and Medicine, James Cook University, Townsville, Queensland, Australia

[2] College of Public Health, Medical and Veterinary Sciences, James Cook University, Townsville, Queensland, Australia

[3] Institut de Biologie Intégrative de la Cellule (I2BC), CEA, CNRS, Université Paris-Sud, Université Paris-Saclay, Gif-sur-Yvette, Orsay, France

[4] Balimo District Hospital, Balimo, Western Province, Papua New Guinea

[5] Department of Medicine, Royal Melbourne Hospital, University of Melbourne, Melbourne, Victoria, Australia

[¤] Current address: National Animal Disease Center, Agricultural Research Service, United States Department of Agriculture, Ames 50010, IA, USA

*Corresponding author

Email: vanina.guernier@gmail.com

**Co-senior authors

Table S1: Detailed microscopy ('ZN smear') and molecular results per sample (n = 80). Shared International Types (SIT) and spoligotype lineages and families were assigned according to signatures provided in SITVIT2 and to the latest genome-based lineage designations.

BDH ID	JCU ID	Best Cq value qPCR IS6110	ZN smear	Location	Spoligotype pattern	Lineage (Spol-name)	Sublineage	SIT	*rpoB* sequencing
154 D/S (2)	JCUB-154	23.20	3+	Kimama		L4/T1	L4_OTH	53	WT
163 D/S (2)	JCUB-163	24.05	3+	Sisiami		L4/T1	L4_OTH	172	WT
254 D/S (2)	JCUB-254	18.95	3+	Kimama		L4/T1	L4.3 or L4.4	4	WT
134 NaOH (2)	JCUB-134	25.13	Unknown	Kubeai		L4/T1	L4.3 or L4.4	4	WT
038 D/S (2)	JCUB-038	21.35	3+	Balimo		L4/T1	L4.2?	absent SITVIT2	WT
061 NaOH (2)	JCUB-061	21.73	3+	Kotale		L4/T1	L4.2?	absent SITVIT2	WT
258 D/S (2)	JCUB-258	21.91	2+,1+	Pisi		L4/T1	L4.2?	absent SITVIT2	WT
250 D/S (2)	JCUB-250	22.06	3+	Pisi		L4/T1	L4.2?	absent SITVIT2	WT
037 D/S (2)	JCUB-037	22.79	3+	Isago		L4/T1	L4.2?	absent SITVIT2	WT
351 D/S (1)	JCUB-351	22.98	3+	Sogere		L4/T1	L4.2?	absent SITVIT2	WT
329 D/S (1)	JCUB-329	23.13	2+	Bamio		L4/T1	L4.2?	absent SITVIT2	WT
359 D/S (1)	JCUB-359	23.93	3+	Kimama		L4/T1	L4.2?	absent SITVIT2	WT
271 D/S (1)	JCUB-271	23.95	3+	Dogono		L4/T1	L4.2?	absent SITVIT2	WT
078 NaOH (1)	JCUB-078	26.37	3+	Kimama		L4/T1	L4_OTH	orphan PNG	WT
305 D/S (1)	JCUB-305	20.87	1+	Pikiwa		L4/T1	L4_OTH	393	WT
319 D/S (1)	JCUB-319	24.22	3+	Pikiwa		L4/T1	L4_OTH	393	WT
367 D/S (1)	JCUB-367	26.94	scanty	Pikiwa		L4/T1	L4_OTH	393	WT
205 NaOH (2)	JCUB-205	27.66	NAFB	Unknown		L4/T1	L4_OTH	393	WT
174 D/S (1)	JCUB-174	20.04	3+	Kaniya		L4/T1	L4_OTH	2952	WT
246 D/S (1)	JCUB-246	25.00	1+	Kaniya		L4/T1	L4_OTH	2952	WT
287 D/S (1)	JCUB-287	23.30	3+	Balimo		L4/T1	L4_OTH	1916	WT
235 D/S (2)	JCUB-235	20.88	3+	Aduru		L4/H1	L4.1.2	47	WT
204 D/S (1)	JCUB-204	21.02	3+	Unknown		L4/H3	L4.1.2 or L4.5	50	WT
237 D/S (1)	JCUB-237	25.41	1+	Kawiyapo		L4	L4_OTH	orphan PNG	WT
283 NaOH (1)	JCUB-283	27.67	2+	Unknown		unknown	possible mixed L2/L4	orphan PNG	WT
310 NaOH (1)	JCUB-310	27.58	1+	Kawiyapo		unknown	possible mixed L2/L4	orphan PNG	WT
331 NaOH (1)	JCUB-331	28.75	1+	Adiba		unknown	possible mixed L2/L4	orphan PNG	WT
187 D/S (2)	JCUB-187	26.99	2+	Saweta		unknown	possible mixed L2/L4	orphan PNG	WT
077 D/S (2)	JCUB-077	26.07	2+	Adiba		unknown	possible mixed L2/L4	orphan PNG	WT
221 NaOH (1)	JCUB-221	26.11	scanty	Saweta		unknown	possible mixed L2/L4	orphan PNG	WT
357 D/S (1)	JCUB-357	26.45	3+	Pisi		unknown	possible mixed L2/L4	orphan PNG	WT
247 D/S (2)	JCUB-247	26.50	1+	Kotale		unknown	possible mixed L2/L4	orphan PNG	WT
244 NaOH (2)	JCUB-244	27.40	2+	Kuria		unknown	possible mixed L2/L4	orphan PNG	WT
032 D/S (2)	JCUB-032	28.01	1+	Emeti Station		unknown	possible mixed L2/L4	orphan PNG	Bad sequence
060 D/S (2)	JCUB-060	28.15	1+	Togowa		unknown	possible mixed L2/L4	orphan PNG	WT
266 NaOH (2)	JCUB-266	28.41	NAFB	Sarau (village not known)		unknown	possible mixed L2/L4	orphan PNG	WT
224 NaOH (1)	JCUB-224	28.36	3+	Waligi		unknown	possible mixed L2/L4	orphan PNG	WT
208 NaOH (1)	JCUB-208	26.81	NAFB	Pisi		unknown	possible mixed L2/L4	orphan PNG	WT
200 NaOH (1)	JCUB-200	27.53	NAFB	Yau		unknown	possible mixed L2/L4	orphan PNG	WT
192 D/S (2)	JCUB-192	28.39	scanty	Saweta		unknown	possible mixed L2/L4	orphan PNG	WT
089 NaOH (1)	JCUB-089	27.08	NAFB	Diwame		unknown	possible mixed L2/L4	orphan PNG	WT
217 NaOH (2)	JCUB-217	27.82	2+	Balimo		unknown	possible mixed L2/L4	3080	WT
241 D/S (1)	JCUB-241	27.94	2+	Wasua		unknown	possible mixed L2/L4	3080	WT
057 NaOH (2)	JCUB-057	26.30	3+	Kotale		unknown	possible mixed L2/L4	orphan SITVIT2	WT
353 D/S (1)	JCUB-353	23.91	2+	Maipani		L1/EAI5		1365	WT
274 D/S (2)	JCUB-274	19.09	3+	Panakawa		L2/Beijing	L2	1	S450L
150 D/S (1)	JCUB-150	19.33	3+	Wasua		L2/Beijing	L2	1	WT
306 D/S (1)	JCUB-306	19.35	3+	Saiwase		L2/Beijing	L2	1	WT
326 D/S (1)	JCUB-326	19.53	3+	Kebane		L2/Beijing	L2	1	WT
063 D/S (2)	JCUB-063	20.41	3+	Kotale		L2/Beijing	L2	1	WT
304 D/S (1)	JCUB-304	20.46	3+	Pisi		L2/Beijing	L2	1	WT
199 D/S (1)	JCUB-199	20.51	3+, 1+	Kewa		L2/Beijing	L2	1	WT
206 D/S (2)	JCUB-206	20.53	2+,3+	Yau		L2/Beijing	L2	1	WT
265 NaOH (1)	JCUB-265	20.53	3+	Togowa		L2/Beijing	L2	1	WT
228 D/S (1)	JCUB-228	20.63	3+	Kimama		L2/Beijing	L2	1	WT
267 D/S (2)	JCUB-267	20.71	3+	Balimo		L2/Beijing	L2	1	WT
118 D/S (1)	JCUB-118	20.92	3+	Widama		L2/Beijing	L2	1	S450L
175 D/S (1)	JCUB-175	21.49	2+	Ugu		L2/Beijing	L2	1	WT
121 D/S (2)	JCUB-121	21.76	3+	Togowa		L2/Beijing	L2	1	WT
082 NaOH (1)	JCUB-082	22.73	2+	Kaenewa		L2/Beijing	L2	1	WT
120 NaOH (2)	JCUB-120	22.78	3+	Diwame		L2/Beijing	L2	1	WT
068 NaOH (2)	JCUB-068	22.96	3+	Saweta		L2/Beijing	L2	1	S450L
171 D/S (1)	JCUB-171	23.01	2+	Makapa		L2/Beijing	L2	1	WT
062 D/S (2)	JCUB-062	23.39	3+	Adiba		L2/Beijing	L2	1	WT
358 D/S (1)	JCUB-358	23.89	3+	Widama		L2/Beijing	L2	1	WT
337 NaOH (1)	JCUB-337	23.95	3+	Ugu		L2/Beijing	L2	1	WT
325 D/S (1)	JCUB-325	23.96	3+	Bimaramio		L2/Beijing	L2	1	WT
203 NaOH (2)	JCUB-203	24.01	2+	Wasua		L2/Beijing	L2	1	WT
361 D/S (1)	JCUB-361	24.35	3+	Balimo		L2/Beijing	L2	1	S450L
028 NaOH (2)	JCUB-028	24.36	NAFB	Dogono		L2/Beijing	L2	1	WT
131 D/S (3)	JCUB-131	24.40	3+	Balimo		L2/Beijing	L2	1	S450L
248 D/S (1)	JCUB-248	24.56	3+	Balimo		L2/Beijing	L2	1	S450L
162 D/S (1)	JCUB-162	24.70	3+	Uladu		L2/Beijing	L2	1	WT
321 D/S (1)	JCUB-321	25.17	1+	Unknown		L2/Beijing	L2	1	WT
303 NaOH (1)	JCUB-303	26.38	2+	Awaba		L2/Beijing	L2	1	WT
343 NaOH (1)	JCUB-343	26.54	2+	Unknown		L2/Beijing	L2	1	WT
135 NaOH (2)	JCUB-135	20.64	Unknown	Saweta		L2/Beijing	L2	1	WT
137 NaOH (2)	JCUB-137	23.95	Unknown	Yau		L2/Beijing	L2	1	WT
322 D/S (1)	JCUB-322	24.19	Unknown	Balimo		L2/Beijing	L2	1	S450L
363 D/S (1)	JCUB-363	27.90	Unknown	Kuria		L2/Beijing		255	WT

Full rpoB sequences (147 bp) amplified:

Wild type (WT) ATCAAGGAGTTCTTCGGCACCAGCCAGCTGAGCCAATTCATGGACCAGAACAACCCGCTGTCGGGGTTGACCCACAAGCGCCGACTGTCGGCGCTGGGGCCCGGCGGTCTGTCACGTGAGCGTGCCGGGCTGGAGGTCCGCGACGTG

S450L mutant* ATCAAGGAGTTCTTCGGCACCAGCCAGCTGAGCCAATTCATGGACCAGAACAACCCGCTGTCGGGGTTGACCCACAAGCGCCGACTGTTGGCGCTGGGGCCCGGCGGTCTGTCACGTGAGCGTGCCGGGCTGGAGGTCCGCGACGTG

* the mutation compared to the reference (WT) is highlighted in red
